# How Do Adolescents Manage Information in the Relationship with Their Parents? A Latent Class Analysis of Disclosure, Keeping Secrets, and Lying

**DOI:** 10.1007/s10964-022-01599-0

**Published:** 2022-03-29

**Authors:** Sophie Baudat, Gregory Mantzouranis, Stijn Van Petegem, Grégoire Zimmermann

**Affiliations:** 1 Family and development research center (FADO), Institute of Psychology, University of Lausanne, Lausanne, Switzerland; 2 Food & Human Behavior Lab, Faculty of Psychology, UniDistance Suisse, Brig, Switzerland; 3 Centre de recherche sur le développement, la famille et les systèmes humains (DeFaSy), Université Libre de Bruxelles, Brussels, Belgium; 4 F.R.S.-FNRS Research Associate, Brussels, Belgium

**Keywords:** Adolescent disclosure, Secrets, Lies, Parenting, Alcohol use, Latent class analysis

## Abstract

The use of disclosure and concealment strategies by adolescents in the relationship with their parents may have important implications for their adjustment. Few studies of adolescents’ information management have taken a person-centered approach, yet it is a useful way to understand variations in how they regulate information shared with their parents. This study explored adolescents’ information management constellations with their mothers and fathers, and how these patterns differ in terms of perceived need-supportive parenting, autonomous reasons for disclosure, and problematic alcohol use. Three hundred thirty-two Swiss adolescents (45% female; *M*_age_ = 15.01 years) reported information management strategies used with each parent (disclosure, keeping secrets, lying), perceptions of maternal and paternal need-supportive parenting (involvement, autonomy support, structure), autonomous reasons for disclosure, and problematic alcohol use. Latent class analyses revealed three classes: *Reserved* (37%), *Communicators* (36%), and *Deceptive* (27%). Comparisons across classes showed that adolescents in the *Communicators* class reported the highest levels of parental involvement and autonomy support, as well as autonomous reasons for disclosure. Adolescents in the *Deceptive* class reported the lowest levels of parental involvement and autonomy support, as well as autonomous reasons for disclosure. Associations between classes and problematic alcohol use were also found, such that the likelihood of problem drinking was greater for adolescents in the *Deceptive* class. These findings underscore the importance of continued information sharing with both parents, and underline how a need-supportive parenting context may encourage adolescents to talk voluntarily.

## Introduction

During adolescence, individuals’ needs for autonomy and privacy become salient, leading them to question their parent’s legitimacy to obtain information about certain areas of their lives (Smetana, 2011). This coincides with an increase in time spent with peer groups in which adolescents experiment with risky behaviors (Barnes et al., 2007), particularly alcohol use (Inchley et al., 2020). Revealing this information to parents can be particularly sensitive (Metzger et al., 2020). Thus, adolescents are likely to set boundaries around information about their daily activities, friendships, and whereabouts. These boundary shifts lead to profound transformations in parent-child communication patterns, as illustrated by adolescents’ tendency to hide more information about their daily lives over time (e.g., Keijsers & Poulin, 2013). Intentionally withholding information from parents is thus assumed to be a part of development, as it would help adolescents gain a sense of autonomy (Smetana, 2018) and assert their privacy within the family (Finkenauer et al., 2008). Despite this assumption, researchers have rarely been able to empirically find positive correlates of adolescents’ concealment. Indeed, adolescents’ frequent withholding of information has been shown to be not only symptomatic of poorer parent-child relationships, which may undermine their motivation to reveal information (Wuyts et al., 2018), but also to have consequences for adolescents’ alcohol use (McCann et al., 2016). To disentangle this apparent paradox, it seems important to simultaneously consider the extent to which adolescents withhold information from their parents and the extent to which they reveal it (Talwar & Crossman, 2011). Person-centered approaches may be particularly useful in this regard to explore distinct patterns of information management and their differences with respect to parenting, motivation to disclose, and alcohol use. However, little research has been conducted with this analytical approach. Using latent class analysis, this study aims to explore the extent to which adolescents vary in their frequency of use of strategies, and to understand how these patterns differ in terms of perceived parenting, autonomous reasons for disclosure, and problematic alcohol use.

### Disclosing, Keeping Secrets and Lying to Parents During Adolescence

Since the pioneering work of Stattin and Kerr (Stattin & Kerr, 2000; Stattin et al., 2010), adolescents’ status as active agents in the relationship with their parents has been widely recognized. Indeed, adolescents actively manage what their parents know about their daily activities, friendships and whereabouts outside the family, strategically selecting the amount and the type of information they communicate (Tilton-Weaver & Marshall, 2008). In this process, adolescents are likely to use a variety of strategies to manage the content of information about their unsupervised time (e.g., Cumsille et al., 2010). *Disclosure* refers to the act of sharing information about their daily life to their parents, including their activities, whereabouts, and the peers with whom they share their time (Tilton-Weaver et al., 2013). *Keeping secrets* from their parents is a way for adolescents to hide information about certain areas of their lives, such as omitting certain details in conversations (Frijns & Finkenauer, 2009). Finally, *lying* is also a concealment strategy that adolescents may use, but it differs from the previous one because it involves intentionally sharing false information to deceive their parents—an act of commission (Smetana, 2011).

Although the strategies of disclosure and concealment (i.e., keeping secrets and lying) are often seen as opposites on the same continuum, these acts of information management are distinct from each other both theoretically (Afifi et al., 2007) and empirically (e.g., Jäggi et al., 2016). Theoretically, keeping secrets and lying require a conscious control decision to hide information, whereas nondisclosure does not require such an effort (Frijns et al., 2010). In this sense, keeping secrets and lying can be considered as intentional ways of managing privacy (Afifi et al., 2007). Moreover, keeping secrets and disclosing information can occur simultaneously (Finkenauer et al., 2008), such as when adolescents tell their parents that they went to a party where their friends drank alcohol, without mentioning that they also drank alcohol (Tasopoulos-Chan et al., 2009). Previous empirical research provides evidence for this theoretical distinction, as studies using factor analysis have confirmed that disclosure and keeping secrets should be considered as two separate constructs (e.g., Jäggi et al., 2016). Furthermore, according to communication researchers (Afifi et al., 2007), keeping secrets should also be distinguished from lying, in that the secret keeper withholds certain information with the intention of making no particular impression on the interlocutor. By contrast, when lying, one seeks more explicitly to create a false impression. Overall, both theoretical and empirical evidence supports the importance of studying these three information management strategies as distinct constructs.

These strategies can take on important developmental functions as adolescents grow older. During this period, fundamental changes occur in the way adolescents manage the information they give their parents about their daily lives (Lionetti et al., 2019). Specifically, adolescents are likely to disclose less information (Laird et al., 2013) and keep more secrets from their parents (Baudat et al., 2020). According to the communication privacy management theory (Petronio, 2002, 2010), such information retention would facilitate the development of adolescents’ autonomy and privacy within their families, as it allows them to regulate and choose the information they share with their parents, and to delimit boundaries around information (Finkenauer et al., 2009). Yet at the same time, along with this need, adolescents also try to keep a sense of intimacy, which involves the need to feel connected and keep good relationships with their parents who remain an important source of support. This may be done through continued disclosure of information about certain aspects of their daily life (Fletcher & Blair, 2018). In summary, it is assumed that adolescents use both disclosure and concealment strategies with their parents to satisfy their needs for autonomy and for intimacy.

### Information Management and Need-Supportive Parenting Style

According to self-determination theory (SDT; Ryan & Deci, 2017), all human beings are born with three basic and universal psychological needs – autonomy, relatedness, and competence – that are essential for well-being and adjustment. Adolescents’ development would be enhanced if they feel autonomous (i.e., feeling a sense of being at the origin of one’s actions and choices), related (i.e., feeling socially connected to significant others), and competent (i.e., feeling a sense of efficacy and mastery). Parents can actively support the satisfaction of these needs through their parenting behaviors (Joussemet et al., 2008). In particular, three components of parenting have been identified as need-supportive in the sense that they provide the essential conditions to satisfy adolescents’ basic psychological needs (Soenens et al., 2019): (a) involvement, which refers to the emotional resources (e.g., warmth, affection, respect) offered by parents to their child (Grolnick, 2003); (b) autonomy support, which concerns the degree to which parents encourage their children to think and behave according to their interests, personal values and goals (rather than by forcing them to behave in a specific way [i.e., controlling parenting]; Soenens et al., 2007); and (c) structure, which refers to clear and consistent expectations, limits and rules communicated to the child (Grolnick & Pomerantz, 2009).

Previous research has shown that need-supportive parenting promotes adolescent development and interpersonal functioning (Pinquart, 2017). With respect to adolescent information management process, many studies showed that adolescents who are more likely to disclose information to their parents about their daily life are those who perceive their mothers (e.g., Kearney & Bussey, 2015) or fathers (e.g., Soenens et al., 2006) as warm and responsive. Adolescents whose parents are autonomy-supportive are also more likely to disclose information about a variety of topics, including mistakes at school (Roth et al., 2009), activities and whereabouts outside the home (Mageau et al., 2017), or friends (Wuyts et al., 2018). Conversely, adolescents who perceive their parents as unresponsive (e.g., Tokić Milaković et al., 2018) or controlling (e.g., Soenens et al., 2006) are less likely to disclose information. Lastly, regarding the links between parental structure and adolescent disclosure, the results are sparser. On the one hand, some studies showed that adolescents whose mothers (e.g., Kearney & Bussey, 2015) or fathers (e.g., Soenens et al., 2006) provide them rules and limits are more likely to disclose information to them. In contrast, other studies found no statistically significant associations between structure and disclosure (e.g., Hamza & Willoughby, 2011).

Previous studies have also examined associations between the dimensions of parenting style and adolescents’ use of concealment strategies, but to a relatively lesser extent than for disclosure. In general, empirical evidence showed that adolescents with need-supportive parents are less likely to keep secrets or lie. For example, perceived parental involvement has been negatively related to keeping secrets (e.g., Keijsers et al., 2010). In addition, perceived parental autonomy support was negatively correlated with lies across multiple topics (Bureau & Mageau, 2014), whereas perceived controlling parenting was positively associated with keeping secrets (Baudat et al., 2020). Similarly, adolescents from authoritative families (i.e., characterized by responsiveness, autonomy support and structure) are less likely to keep secrets (Almas et al., 2011) or to lie (Darling et al. 2006). Finally, as presented above for disclosure, the associations between structure and concealment strategies are also less consistent. For example, some studies showed that adolescents whose parents set rules and limits (e.g., Jensen et al., 2004) were more likely to lie, whereas others have found no statistically significant links between structure and lying (e.g., Lushin et al., 2017).

Taken together, these findings suggest that the general parenting style adopted by parents is important in understanding how adolescents manage information. Specifically, adolescents who are most likely to disclose information have parents who are involved, autonomy-supportive, and, to some extent, structuring. Thus, parents also have a role to play in the information management process by creating a need-supporting climate in which adolescents are willing to share information about their daily lives. Adolescents are indeed likely to interpret cues in their family context that satisfy their needs (Tokić Milaković et al., 2018) and in turn, may foster their motivation to disclose information (Wuyts et al., 2018).

### Underlying Reasons for Information Management

Previous studies mainly examined the extent to which adolescents disclose or conceal information. However, to date, the voluntary or involuntary nature of that disclosure has received little attention. Drawing on SDT (Ryan & Deci, 2017), one study (Wuyts et al., 2018) differentiated willingness to disclose by examining underlying reasons, which vary along a continuum from controlled to more autonomous motivations. When adolescents disclose information to their parents because they feel pressured to do so, whether by an external or internal cause, they disclose because of *controlled* reasons. The most pressured reasons involve “external regulation”, such as when an adolescent reveal information out of fear of punishment or disappointment of parents. Pressure can also come from within (“introjected regulation”), for example when adolescents disclose information because they would feel ashamed or guilty if they did not. Conversely, adolescents may also disclose information because they want to; that is, for *autonomous* reasons. Specifically, in the case of “identified regulation”, adolescents disclose information to their parents because they personally understand that it is important to be honest in the relationship with them. Finally, in the case of “intrinsic motivation”, adolescents disclose information to their parents because they find it interesting to talk with them and enjoy doing so.

### Information Management and Problematic Alcohol Use

As adolescents spend more time with their peers, they may engage more frequently in risky behaviors (e.g., substance use, delinquency, unsafe sex; Barnes et al., 2007). One of the most common behaviors among adolescents is alcohol use (Inchley et al., 2020). For example, two thirds of Swiss 15-year-old students report having consumed alcohol at least once in their lifetime (70.3% of boys and 68.5% of girls) (Delgrande Jordan et al., 2019). In addition, nearly one in two 15-year-old students report drinking alcohol in the 30 days prior to the survey (46.0% of boys and 41.0% of girls). These behaviors are generally explorative (Michaud, 2006) and may have a developmental function (Zimmermann et al. 2017). What remains a legitimate concern is the significant proportion of adolescents involved in excessive alcohol use (Delgrande Jordan et al., 2019). Adolescent problem drinking patterns can have short- and long-term deleterious consequences (Boden & Fergusson, 2011), such as risky sexual behavior (Wu et al., 2010) or depression (Mason et al., 2008). Therefore, public health actors and researchers are working to identify factors that help understand and prevent the onset of these behaviors (e.g., Alcohol and Public Policy Group, 2010).

Previous research suggests that adolescents’ information management may be an important aspect to consider in the prevention of alcohol use. Specifically, negative associations have been found between disclosure to mothers and fathers and frequency of alcohol use (Jiménez-Iglesias et al., 2013), whereas positive associations have been found between keeping secrets and hazardous drinking (Baudat et al., 2020) as well as alcohol-related mental or physical health problems (Hartman et al., 2015). It should be noted that these cross-sectional associations could potentially reflect the fact that adolescents’ information management not only contributes to and reinforces certain behavioral patterns, but is also shaped by these behavioral patterns (Marshall et al., 2005). For example, longitudinal studies have shown that higher levels of disclosure predicted less hazardous drinking over time (Stavrinides et al., 2010), whereas higher levels of lying predicted more frequent drinking (Lushin et al., 2017). In addition, bidirectional associations were also found, with lower levels of kept secrets being associated with less frequent drinking over time, and frequent drinking being associated with greater subsequent secrets (McCann et al., 2016).

Considering the normative aspect of concealment in adolescence discussed above, some authors have concluded that adolescents should use concealment strategies sparingly in order to promote their development as well as satisfactory relationships with their parents (e.g., Talwar & Crossman, 2011). In this sense, distinguishing patterns of disclosure, keeping secrets, and lying, and examining whether these patterns differ with respect to adolescents’ drinking could be a key element to understanding the paradox between “the dark side and light side” (Afifi et al., 2007, p. 61) of information management. Person-centered approaches may be particularly useful in this respect to better understand the differences in information management patterns with respect to parenting and adolescent drinking.

### Person-Centered Approaches to Adolescent Information Management

Recent works in the field of developmental psychology have taken a new turn in examining the diversity of family experiences during the adolescent period by using person-centered approaches (Frijns et al., 2020). Whereas variable-centered approaches, which are predominantly employed in this literature, examine associations between variables and assumes that these links are similar across families, person-centered approaches allow identifying whether subgroups of similar subjects exist within a population (Howard & Hoffman, 2017). With respect to adolescents’ information management, these approaches have the advantage of providing a more nuanced view of the process of information management by identifying different subgroups of adolescents based on their use of disclosure, keeping secrets, and lying, and how these distinct patterns differ with respect to the quality of the parent-child relationship (Rote & Smetana, 2018) and adolescent outcomes (Elsharnouby & Dost-Gözkan, 2020).

To date, only a limited number of studies have used a person-centered approach to investigate the unique ways in which adolescents regulate information shared with their parents. Moreover, the few existing studies have focused on adolescents’ use of disclosure or keeping secrets (Elsharnouby & Dost-Gözkan, 2020) in the relationship with their mother specifically (Cumsille et al., 2010) or without distinguishing between mother and father (Padilla-Walker et al., 2018). For example, one longitudinal study (Padilla-Walker et al., 2018) showed that the majority of adolescents (82%) experienced a gradual decrease in mean levels of disclosure to parents over the course of adolescence, while another group reported low and stable levels of disclosure (13%), and the third group reported a steep decrease in disclosure (5%). Group membership was also associated with perceived parenting and adjustment, with adolescents in the second profile (low-stable) reporting higher levels of delinquency, lower levels of prosocial behaviors toward the family, and lower levels of perceived maternal warmth. Given that adolescents generally disclose more information to their mothers than to their fathers (e.g., Smetana et al., 2006), a joint examination of disclosure and concealment strategies used by adolescents in relationships with their mother and their father would be informative. Extending this work by considering both disclosing information to and keeping secrets from mother, father and best friends, a recent study (Elsharnouby & Dost-Gözkan, 2020) highlighted five information management profiles: Highest Disclosure to Parents-Lowest Secrecy; Average Disclosure and Secrecy; Low Disclosure-High Secrecy; Low Disclosure-Highest Parent Secrecy; Lowest Disclosure-Low Secrecy. Adolescents in profiles characterized by low mean levels of disclosure and by high mean levels of secret keeping reported low levels of well-being (i.e., low life satisfaction, low confidence in problem-solving, and high anxiety). However, this study did not include the lying strategy in the examination of information management profiles, nor did it examine whether profiles differ in terms of need-supportive parenting, autonomous reasons to disclose information, or alcohol use.

## Current Study

Few studies have taken a person-centered approach to identify adolescents’ patterns of disclosure, keeping secrets, and lying in the relationship with their mothers and fathers, which limits understanding of the different ways in which adolescents regulate the information shared with their parents and how these patterns differ from one another. The first aim of the present study was thus to identify classes of information management among adolescents based on the frequency of use of disclosure, keeping secrets, and lying with their mother and their father. Based on previous research, at least two different classes of information management have been hypothesized. Specifically, one group was expected to frequently disclose information to their mother and father and rarely or never keep secrets or lie, whereas a second group was expected to be characterized by the opposite pattern of frequently keeping secrets and lying with their mother and their father and relatively rare use of disclosure. Although we did not have explicit expectations, groups with more mixed patterns (e.g., occasional use of all strategies in the relationships with both parents, or a combination of frequent use of disclosure with their one parental figure and occasional use of disclosure with the other parental figure) could emerge as well. The second aim was to examine the extent to which these patterns differ with respect to the dimensions of need-supportive parenting, autonomous reasons for disclosure, and problematic alcohol use. Based on previous studies, it was expected that adolescents in the classes characterized by frequent disclosures as well as rare secrets and lies would perceive more need-supportive parenting than adolescents in other classes (i.e., those characterized by frequent secrets and lies or mixed patterns). They would also report more autonomous reasons for disclosure and would be less involved in problematic alcohol use than adolescents in other classes.

## Methods

### Participants and Procedure

The present cross-sectional study was conducted in three public schools (one urban, one semi-urban, and one rural) in the French-speaking part of Switzerland. A few weeks before data collection, adolescents in their last year of compulsory school (*M*_age_ = 15.01, *SD* = 0.72) and their families were informed about the objectives of the study and the confidential treatment of the data. Because the risk of participating in this study was very limited, oral informed consent was obtained from the adolescents and passive informed consent was obtained from their parents; that is, parents who did not want their adolescents to be involved in the study could express their refusal by completing and returning a non-consent form. A total of 449 families were contacted, of which 71 declined to participate, and 27 adolescents were absent on the day of data collection (response rate = 78%). Three hundred and fifty-one adolescents who agreed to participate in the study were asked to complete a series of self-report questionnaires in their classroom, under the supervision of the first author and a graduate student from the research team.

Because this study aimed to simultaneously examine information management strategies in the mother-teen and father-teen dyads, participants without both parents (*n* = 19) were excluded from the analyses. The final sample was composed of 332 adolescents (45% female). Most of them had a Swiss (68.6%) or European (24.6%) nationality. Most participants lived in a family with both parents living together (74.4%). About half of the participants followed a general-oriented education (53.7%) and the other half followed an academic-oriented education (46.3%). Compared to the financial situation of other families in Switzerland, most participants perceived their own situation as average (62.7%).

### Measures

All questionnaires were already available in French or translated through a back-translation procedure. Participants were asked about their information management strategies and perceptions of need-supportive parenting for each parent separately.

#### Information management strategies

Three distinct information management strategies were assessed: disclosure, keeping secrets, and lies. Adolescents’ responses were rated on a 5-point Likert-type scale (1 = *never*, 2 = *rarely*, 3 = *occasionally*, 4 = *often*, 5 = *always*). Consistent with previous research (e.g., Frijns et al. 2010), disclosure and secret keeping were assessed separately using the Child Disclosure Scale (Stattin & Kerr, 2000). The disclosure scale (three items) evaluates how often adolescents disclose to their mother/father information about school and leisure activities (e.g., “I spontaneously tell my parents about my friends [which friends I hang out with and how they think and feel about various things]”). As in previous studies (e.g., Keijsers et al., 2009), this scale had acceptable internal consistency, given its small number of items (α = 0.60 for mothers/α = 0.63 for fathers). The secrets scale (two items) measures how often adolescents keep secrets from their mother/father (e.g., “I keep much of what I do in my free time secret from my parents”). As previous research has shown (Keijsers & Poulin, 2013), this scale also demonstrated an acceptable consistency (α = 0.66/0.65). Finally, adolescents’ explicit lying to their mother/father was assessed with an adapted version of a lying scale developed by Engels et al. (2006). Four items assessing how often adolescents explicitly lie to their parents about their activities and actions were used (e.g., “I lie to my parents about what I do with my friends”). Internal consistency was good (α = 0.77/0.77).

#### Need-supportive parenting

Consistent with SDT (Soenens et al., 2019; Vansteenkiste & Ryan, 2013), we assessed three dimensions characteristic of need-supportive parenting: involvement, autonomy support and structure. Adolescents’ responses were rated on a 5-point Likert-type scale (1 = *totally disagree*, 5 = *totally agree*). First, involvement was measured using the seven-item Acceptance-Rejection subscale from the Child Report of Parent Behavior Inventory (CRPBI; Schaefer, 1965; Schludermann & Schludermann, 1988). This scale assesses the degree to which parents are perceived as involved, responsive, and loving (e.g., “My parents believe in showing their love for me”). In line with previous studies (e.g., Albert Sznitman et al., 2019), the internal consistency was good (*α* = 0.86/0.88). Second, autonomy support was assessed with the autonomy support subscale (12 items) of the Perceived Parental Autonomy Support Scale (P-PASS; Mageau et al., 2015). This subscale evaluates the extent to which parents offer choice within certain limits (four items), provide rationale for requests and rules (four items), and acknowledge their adolescents’ feelings (four items). An example item is: “My parents were open to my thoughts and feelings even when they were different from theirs”. As previous studies have shown (Bureau & Mageau, 2014; Joussemet et al., 2018), this scale had a good internal consistency (*α* = 0.88/0.88). Third, structure was assessed using the Parental Control Scale (Stattin & Kerr, 2000). This scale specifically evaluates the extent to which parents set rules and limits regarding their adolescents’ disclosure of their free time (e.g., “Before you go out on a Saturday evening night, do you have to tell your parents where you are going and with whom?”). Therefore, this questionnaire differs from other measures developed in the SDT, which assess structure more broadly, measuring the degree of clarity and consistency of rules and expectations, predictability of consequences, provision of rationales, and parental authority (Grolnick et al., 2014). Like previous studies (e.g., Keijsers et al., 2009), the scale demonstrated good internal consistency (*α* = 0.80/0.83).

#### Adolescents’ reasons for disclosure

Adolescents’ autonomous (vs. controlled) reasons for disclosure were measured by a version of the Self-Regulation Questionnaire (SRQ; Ryan & Connell, 1989) adapted to the context of adolescent information management (Wuyts et al. 2018). This 24-item scale began with the sentence, “When I talk to my parents about the things I do, it’s because…”. Next, adolescents rated the items tapping into different reasons. In line with SDT (Ryan & Deci, 2017), four types of regulation were assessed: external regulation (6 items; “…otherwise I will be punished”), introjected regulation (6 items; “…I would feel guilty if I would not do so”), identified regulation (6 items; “…doing so matches with my values”), and intrinsic regulation (6 items; “…talking to my parents about what I do is pleasant”). Responses were rated on a 5-point Likert scale (1 = *totally disagree*, 5 = *totally agree*). All subscales demonstrated good internal consistency (α = 0.82, 0.73, 0.84, 0.92 for external, introjected, identified, and intrinsic subscales, respectively). In line with previous studies (e.g., Wuyts et al., 2018), a Relative Autonomy Index (RAI) was calculated by weighting the four types of regulation according to their position on the self-determination continuum. Specifically, external, introjected, identified and intrinsic subscales were respectively weighted −2, −1, +1, and +2, and then the weighted scores were summed. Thus, the maximum score was +12 and the minimum score was −12. Higher scores on the RAI suggest more autonomous and less controlled reasons for disclosure. In other words, when adolescents score high on the RAI, they are more likely to disclose information to their parents because they personally want to rather than because they feel forced to do so.

#### Problematic alcohol use

The French version of the Alcohol Use Disorder Identification Test (AUDIT; Saunders et al., 1993) was used to assess problematic alcohol use among adolescents. Although the AUDIT is a clinical instrument used primarily in the adult population, previous studies have demonstrated its usefulness in screening for alcohol use problems in adolescents (e.g., Liskola et al., 2021). This questionnaire consists of 10 items, the first eight covering the past 12 months and the last two covering the entire lifetime. The questions assess alcohol use (Questions 1 through 3), alcohol use disorder (Question 4 through 6), and alcohol-related problems (Questions 7 through 10). Each question is scored from 0 to 4 points, for a maximum sum score of 40. As in previous studies (e.g., Liskola et al., 2018), if a participant scored “0” on the first question and did not answer the other questions, indicating that he or she did not consume alcohol, a total score of zero was given. In a relatively large study population consisting of clinical and school samples (Liskola et al., 2018), the optimal cut-off value found to detect problematic alcohol use in adolescents was ≥ 5. Therefore, consistent with this literature (Liskola et al., 2021), participants who scored < 5 were identified as “AUDIT-negative” and those who scored ≥ 5 were identified as “AUDIT-positive”.

### Statistical Analysis

All statistical analyses were performed using R software (version 4.0.2; R Core Team, 2020). There were 3.9% of missing data. The missing data were replaced using the regularized iterative Principal Components Analysis (PCA) algorithm described by Josse and Husson (2012, 2016). The advantage of this imputation method is that it simultaneously considers the similarities between individuals as well as the links between variables. This procedure was applied for all measures except for the AUDIT. Indeed, as recommended in previous studies (e.g., Liskola et al., 2018), when the item other than the first question was missing, the missing item was imputed with the mean of the completed items. When more than one item was missing, the questionnaires were skipped (*n* = 9).

To identify patterns of information management strategies, latent class analyses (LCA; McCutcheon, 1987) were conducted using the “poLCA” package (Linzer & Lewis, 2011). The interest of LCA is to identify and describe qualitatively different subgroups within a population (latent classes) based on the interrelation between participants’ responses to categorical response variables. LCA was preferred over latent profile analysis (LPA) because it is applied to categorical response variables and because the variables of lying to mother and father tended to be positively skewed. In total, three strategies of information management for each parent (i.e., disclosure to mother/father, keeping secrets from mother/father, lying to mother/father) were used together to identify latent classes. Adolescents reports of information management strategies were rounded to indicate the frequency of use.

First, a series of models were estimated, starting with a one-class model, and then specifying models with an additional class until the model provided no further statistical and conceptual improvement (Nylund-Gibson & Choi, 2018). To ensure that the best solution was found, we set the maximum number of iterations to 5000 and used 50 different sets of starting values (Linzer & Lewis, 2011; Oberski, 2016). Following recommendations in the literature (e.g., Nylund-Gibson & Choi, 2018; Weller et al., 2020), we compared LCA models with different numbers of latent classes and selected the optimal solution based on multiple statistical fit indices coupled with the theoretical interpretability of classes. Specifically, the following statistical information criteria (IC) were used: Bayesian Information Criterion (BIC), Akaike Information Criterion (AIC), sample-size adjusted BIC (SABIC), and Consistent AIC (CAIC), with lower IC scores indicating superior fit. An elbow plot was also used to facilitate examination of model fit (Nylund-Gibson & Choi, 2018). In addition to IC, we compared models with an adjacent number of classes with the Lo-Mendell-Rubin adjusted likelihood ratio test (LMR-LRT). A statistically significant result indicates that the inclusion of an additional class improves model fit (Bauer, 2021). After assessing model fit, we examined several classification diagnostics, although these were not intended to select a final model (Masyn, 2013), but rather to examine the accuracy of classification. Specifically, we considered the entropy, with a value close to 1 and greater than 0.80 supporting the accuracy of the model classification (Nylund-Gibson & Choi, 2018). Average posterior class probabilities (AvePP) were also considered to examine the average probability that the LCA model correctly classifies participants into their most likely class, with values above 0.90 being ideal (Muthén & Muthén, 2000) and those between 0.80 and 0.90 being acceptable (Weller et al., 2020). Also considered was the number of participants in each class, which should not contain fewer than 50 cases or 5% of the sample (Muthén & Muthén, 2000), unless the model fit strongly supported the chosen solution and the small class makes theoretical sense (Weller et al., 2020). Once the optimal model was identified, we interpreted and labeled the classes by examining the response probabilities conditional on class membership, that is, the probability of an individual choosing a specific response category for a given manifest variable as a function of class membership (e.g., the probability of an adolescent answering “often” for the manifest variable “disclosure” given his/her membership in a certain information management class). Each participant was assigned to a specific class based on their posterior class membership probabilities.

Finally, a series of analysis of variance (ANOVA), followed by post hoc analyses based on Tukey’s HSD test, were conducted to examine the extent to which information management classes differed with respect to adolescents’ perceptions of parental involvement, autonomy support and structure as well as autonomous reasons for disclosure. A chi-square test, followed by pairwise comparison analyses, was conducted to examine the association between class membership and adolescent problematic alcohol use. To further explore this association, additional analyses involved testing logistic regression models predicting problem drinking by class.

## Results

As shown in Table 1, descriptive statistics for the study variables suggested that, on average, adolescents often disclose information to their mother, occasionally to their father, and rarely keep secrets from or lie to both parents. In addition, adolescents reported relatively high levels of involvement, autonomy support, and structure on average from both parents. Moreover, adolescents reported on average disclosing information for autonomous reasons. Finally, one in four adolescents was identified as AUDIT-positive.

**Table 1:** Descriptive statistics (Means [Standard Deviation] and Proportions) of study variables

	Parents	
	Mother	Father
Information management strategies		
Disclosure	3.62 (0.88)	3.12 (0.95)
Kept secrets	2.24 (1.08)	2.34 (1.10)
Lies	1.96 (0.90)	1.99 (0.90)
Perceived need-supportive parenting		
Involvement	4.11 (0.69)	3.82 (0.81)
Autonomy support	4.04 (0.65)	3.89 (0.68)
Structure	4.32 (0.79)	4.13 (0.93)
Autonomous reasons for disclosure	3.83 (3.26)	
AUDIT-positive	25.1%	

### Latent Class Analysis

**Fig. 1:**
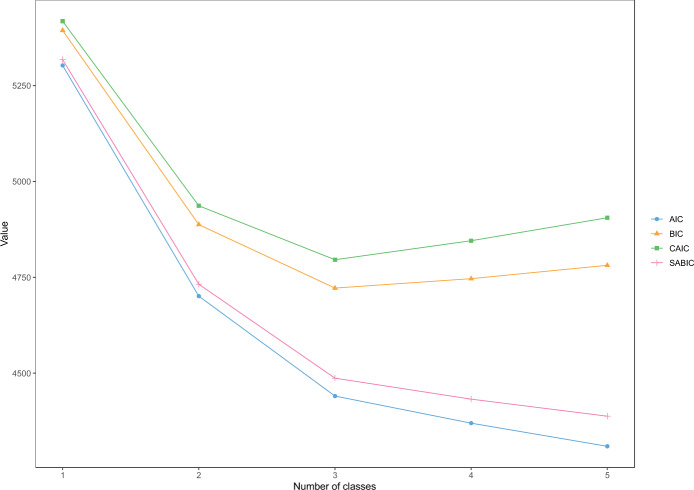
Elbow-Plot showing fit indices for LCA models with increasing number of classes

Latent class models with increasing numbers of classes (1 to 5) were estimated and compared until there was no further statistical and theoretical improvement. Table 2 shows the statistical fit indices and diagnostic criteria for the LCA models. Figure 1 presents an elbow plot to help interpret model fit. As is often the case in LCA models, not all fit indices supported a single solution (Nylund-Gibson & Choi, 2018). Therefore, based on the BIC, which is considered the most reliable indicator (Nylund et al., 2007), and the theoretical interpretation of the classes, the three-class model was selected. For the classification diagnostics, the entropy of the three-class model was 0.98, supporting the accuracy of the model classification. The average posterior probabilities were 0.989 for Class 1, 0.997 for Class 2, and 0.999 for Class 3, indicating accurate prediction of class membership for individuals. Enough participants were found in each class (Class 1: *n* = 124; Class 2: *n* = 119; Class 3: *n* = 89).

**Table 2:** Latent class model fitting and diagnostic criteria

Model	Model fit criteria									
	LL	df		BIC	AIC		SABIC	CAIC		LMR-LRT
1 class	−2627.31	308		5393.94	5302.62		5317.81	5417.94		<0.001
2 classes	−2301.51	283		4887.48	4701.03		4732.05	4936.48		<0.001
**3 classes**	−**2146.18**	**258**		**4721.94**	**4440.36**		**4487.21**	**4795.94**		**<0.001**
4 classes	−2085.90	233		4746.50	4369.80		4432.47	4845.50		<0.001
5 classes	−2030.76	208		4781.36	4309.52		4388.03	4905.36		<0.001

Model	Classification diagnostics									
	Smallest class count (*n*)		Smallest class size (%)			Entropy			Smallest AvePP	
1 class	332		100			–			–	
2 classes	113		34			0.92			0.98	
**3 classes**	**89**		**27**			**0.98**			**0.99**	
4 classes	53		16			0.98			0.95	
5 classes	44		13			0.98			0.95	

*Note*. *LL* LogLikelihood, BIC bayesian information criterion, *AIC* akaike information criterion, *SABIC* sample-size adjusted BIC, *CAIC* consistent AIC, *LMR-LRT* Lo-Mendell-Rubin adjusted likelihood ratio test, *AvePP* average posterior class probabilities. Classification diagnostics are presented for information but should not be used as model selection statistics (Masyn, 2013). The bold font indicates the selected model

**Fig. 2:**
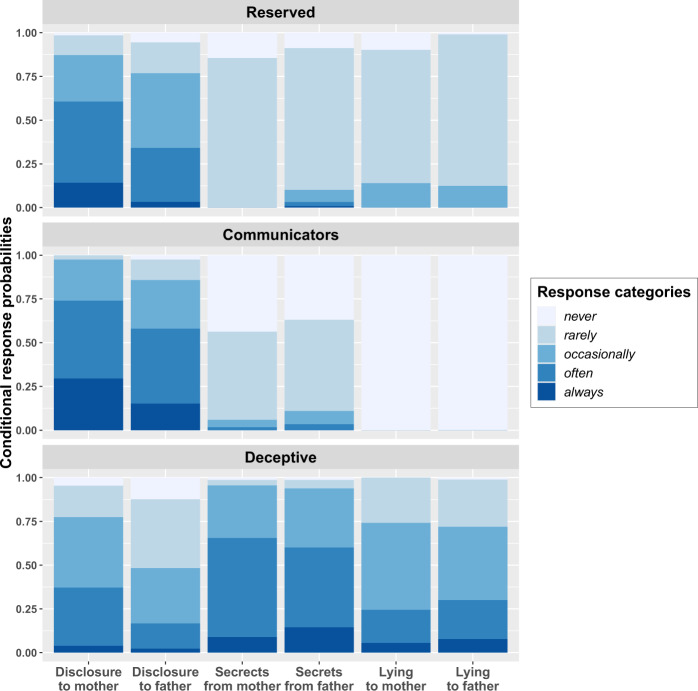
Conditional response probabilities

Then, each latent class was interpreted and labeled by examining the conditional item-response probabilities (see Fig. 2). The first class, labeled *Reserved* (37% of the total sample), consisted of participants who had moderate probabilities of often disclosing information to their mother (probabilities of responding to the response category *often*: 0.46) and occasionally to their father (probabilities of responding to the response category *occasionally*: 0.43). Members of this class also had high probabilities of rarely keeping information secret from and lying to their mother (probabilities of responding to the response category *rarely*: 0.86/0.76) and their father (0.81/0.87). The second class, labeled *Communicators* (36%), comprised participants who had moderate probabilities of often disclosing information to their mother and father (probabilities of responding to the response category *often*: 0.45/0.43, respectively). Members of this class also had moderate probabilities of rarely keeping secrets from their mother and father (probabilities of responding to the response category *rarely*: 0.50/0.52, respectively). Members of this class showed a high likelihood of never lying to their mother and father (probabilities of responding to the response category *never*: 1.00/1.00, respectively). Finally, the third and smallest class, labeled *Deceptive* (27%), included participants who had moderate probabilities of occasionally disclosing information to their mother (probabilities of responding to the response category *occasionally*: 0.40) and moderate probabilities of rarely disclosing to their father (probabilities of responding to the response category *rarely*: 0.39). Members of this class also had moderate probabilities of often keeping secrets from their mother and father (probabilities of responding to the response category *often*: 0.57/0.46, respectively), and moderate probabilities of occasionally lying to their mother and father (probabilities of responding to the response category *occasionally*: 0.50/0.42, respectively).

### Differences Between Information Management Classes

The second objective was to examine the extent to which these classes differed with respect to perceived parenting, autonomous reasons for disclosure, and problematic alcohol use. Table 3 presents, for each class, descriptive statistics for the dimensions of need-supportive parenting (i.e., involvement, autonomy support, structure) and autonomous reasons, as well as the proportions of AUDIT-positive adolescents.

**Table 3:** Means (standard deviations) and proportions differences between classes of information management with maternal and paternal figures

Variable	Classes			*F* ratio	χ^2^	*adj. p*-value	Effect sizes
	Reserved	Communicators	Deceptive				
Involvement							
Mother	4.10 (0.62)_a_	4.34 (0.54)_b_	3.83 (0.84)_c_	15.39	–	<0.001	0.09
Father	3.78 (0.79)_a_	4.10 (0.68)_b_	3.51 (0.89)_c_	14.69	–	<0.001	0.08
Autonomy Support							
Mother	4.00 (0.64)_a_	4.31 (0.48)_b_	3.75 (0.71)_c_	22.25	–	<0.001	0.12
Father	3.78 (0.69)_a_	4.20 (0.53)_b_	3.63 (0.68)_a_	23.13	–	<0.001	0.12
Structure							
Mother	4.32 (0.72)	4.39 (0.81)	4.21 (0.83)	1.33	–	0.266	0.01
Father	4.15 (0.86)_ab_	4.26 (0.89)_a_	3.93 (1.04)_b_	3.34	–	0.042	0.02
Autonomous reasons for disclosure (RAI)	3.36 (2.72)_a_	5.65 (3.08)_b_	2.05 (2.98)_c_	41.20		<0.001	0.20
AUDIT-positive	20.8%_a_	8.5%_b_	53.5%_c_	–	55.11	<0.001	0.41

*Note*. *RAI* relative autonomy index. Means and proportions with different alphabetic superscripts within a row significantly differ at *adjusted p* < 0.05. For interpretation of the effect sizes of the ANOVAs, eta squared (η^2^) = 0.01 indicates a small effect, η^2^ = 0.06 indicates a medium effect, and η^2^ = 0.14 indicates a large effect. For interpretation of the effect size of the chi-squared test, a Cramer’s V (V) of 0.1 to 0.3 indicates a weak association, a V of 0.3 to 0.5 indicates a medium association, and a V above 0.5 indicates a strong association

First, a series of analyses of variance (ANOVA) showed statistically significant differences between classes in terms of maternal and paternal involvement, maternal and paternal autonomy support, and paternal structure. No statistically significant differences emerged in terms of maternal structure. As expected, post hoc analyses using Tukey’s HSD test indicated that adolescents in the *Communicators* class perceived their mother and their father as more involved and autonomy-supportive than those in the *Reserved* and *Deceptive* classes. They also reported higher levels of paternal structure than adolescents in the *Deceptive* class. Regarding adolescents in the *Reserved* class, they perceived their mother and father as more involved than adolescents in the *Deceptive* class and reported higher levels of maternal autonomy support.

Second, the ANOVA results also confirmed class differences in autonomous reasons for disclosure. Post hoc analyses using Tukey’s HSD showed that adolescents in this *Communicators* class reported more autonomous reasons for disclosure to their parents than adolescents in the *Reserved* and *Deceptive* classes. Additionally, adolescents in the *Reserved* class reported more autonomous reasons for disclosure than adolescents in the *Deceptive* class.

Third, results of the chi-square test of independence revealed a statistically significant association between class membership and being AUDIT-positive. As expected, post hoc pairwise comparisons showed that adolescents in the *Communicators* class were less likely to be AUDIT-positive (8.5%) than adolescents in the *Reserved* (20.8%) and *Deceptive* (53.5%) classes. In addition, adolescents in the *Deceptive* class were more likely to be AUDIT-positive than adolescents in the *Reserved* class. Because the association between class membership and problem drinking was moderate according to Cramer’s V (V = 0.41), additional analyses were conducted to further explore this relationship. Specifically, two binary logistic regression models were tested to predict adolescents’ problematic drinking based on class membership. The results are presented in Table 4. Overall, adolescents’ class membership was associated with problematic alcohol use. For Model 1 (reference = *Communicators* class), the likelihood to be AUDIT-positive was 2.82 times for adolescents in the *Reserved* class compared to adolescents in the *Communicators* class. Furthermore, adolescents from the *Deceptive* class were even 12.3 times more likely to be AUDIT-positive than those from the *Communicators* class. For Model 2 (reference = *Reserved* class), adolescents in the *Deceptive* class were 4.37 times more likely to be AUDIT-positive compared to those in the *Reserved* class.

**Table 4:** Results from binary logistic regression models of class membership predicting problematic alcohol use

Variable	Estimate	SE	OR	OR 95% CI	adj*. p*-value
Class (ref. Communicators)					
Reserved	1.04	0.40	2.82	1.32–6.43	0.010
Deceptive	2.51	0.40	12.30	5.88–28.00	<0.001
Class (ref. Reserved)					
Communicators	−1.04	0.40	0.36	0.16–0.76	0.010
Deceptive	1.47	0.31	4.37	2.39–8.15	<0.001

*Note*. *SE* standard error, *OR* odds ratio, *CI* confidence interval.

## Discussion

Adolescents’ communication with their parents changes over the course of adolescence, generally with an increase in the use of secrets and lies and a decrease in disclosure. Although these changes are thought to help adolescents gain autonomy and assert their privacy (Finkenauer et al., 2008; Smetana, 2018), previous studies have rarely evidenced positive correlates of adolescents’ use of concealment strategies. Simultaneously examining the frequency of use of disclosure, secrets, and lies could help clarify this apparent paradox. However, to date, relatively little research has taken a person-centered approach to identify the different profiles of information management strategies used by adolescents to communicate with their parents. Using latent class analysis, this study aimed to examine the extent to which adolescents vary in their frequency of use of three different information management strategies (disclosure, keeping secrets, lying) in the relationship with their mother and father. The second objective was to explore the extent to which these classes differed in terms of parenting (i.e., involvement, autonomy support, and structure), autonomous reasons for disclosure, and problematic alcohol use.

### Classes of Disclosure, Secrecy and Lying to Mother and Father

Regarding the first study objective, distinct patterns of information management that reflect differences in the frequency with which adolescents use the strategies of disclosure, keeping secrets, and lying emerged as expected. For two of the three patterns that emerged, adolescents were likely to disclose information to both parents relatively frequently and to keep secrets or lie more rarely. Specifically, adolescents in the *Reserved* class were likely to frequently disclose information to their mother and occasionally to their father, while rarely keeping secrets and rarely lying to both parents. Adolescents in the *Communicators* class, on the other hand, were likely to frequently disclose information to both parents while rarely keeping secrets and never lie. These patterns suggest that the majority of adolescents accept their parents’ involvement in their lives to some degree (Son & Padilla-Walker, 2021), while at the same time keeping some information secret. These observations are consistent with their growing need for autonomy and privacy, which can be satisfied by keeping some information secret (Finkenauer et al., 2009), either to themselves or by sharing it with a confidant other than their parents (Frijns et al., 2013). At the same time, adolescents also need to maintain a close relationship with their parents, which may be achieved through disclosure (Fletcher & Blair, 2018). Although communication remained open in most families, as evidenced by the likelihood of adolescents disclosing information in these two classes, the results also highlight that some adolescents restrain communication with their parents. Indeed, those in the *Deceptive* class tended to hide information by keeping it secret or by lying, and they disclosed less frequently to their parents. In other words, these adolescents were more likely to create boundaries around information about their extra-familial lives.

Adolescents’ information management profiles in the relationship with their mother and father were relatively similar. Nevertheless, one difference that remains interesting to note between the *Reserved* class and the *Communicators* class is the frequency of adolescents’ disclosure to their mother and father. While adolescents in both classes were characterized by a tendency to frequently disclose information to their mother, this was not so much the case in the relationship with their father. This result is consistent with previous studies showing that adolescents are more likely to disclose information to their mothers than to their fathers (Elsharnouby & Dost-Gözkan, 2020). It also echoes the fact that mothers are generally more aware of their adolescents’ behaviors and activities than fathers (Waizenhofer et al., 2004). This difference may reflect the maintenance of traditional parental roles in some families. Indeed, although mothers’ rate of paid employment has increased markedly since the 1970s in OECD countries (OECD, 2010), they still spend significantly more time with their children than fathers do (Offer, 2013), providing them with opportunities to have conversations with their children during which adolescents can share information (Willoughby & Hamza, 2011). Beyond time spent together, mothers also take on more responsibility for disciplining, daily care, and leisure activities for adolescents (Phares et al., 2009). By being involved in more areas of adolescents’ lives, including their interests and emotional well-being, mothers are generally perceived as a source of emotional support and preferred discussion partners (Smetana et al., 2006). This division of roles between mothers and fathers is known to be colored from childhood by gender role beliefs as well as societal and institutional constraints (Pedulla & Thébaud, 2015). For example, previous studies have shown that parents’ endorsement of an egalitarian gender ideology is associated with father warmth and engagement in childcare (Kuo et al., 2018). However, even if parents have an egalitarian view of gender roles, factors related to their environment, such as workplace characteristics, may still limit fathers’ involvement. For example, greater experience with work-family conflict prevents fathers’ engagement (Kuo et al., 2018). In contrast, parental leave for fathers (Petts & Knoester, 2018), job flexibility (Carlson et al., 2021), and family-supportive workplace (Holmes et al., 2020) have been shown to promote father warmth and engagement.

Overall, the results suggest that adolescents actively manage their parents’ access to information through a mixture of disclosure and concealment strategies, as indicated by previous studies (e.g., Elsharnouby & Dost-Gözkan, 2020). These observations underscore the value of examining the unique ways in which adolescents regulate the boundaries of their activities and friendships with both parents by considering multiple information management strategies, rather than examining these separately. To explain the observed variation in adolescents’ information management, we next examined whether these patterns differ with respect to perceptions of parenting and their own drinking behaviors.

### Information Management Classes and Need-Supportive Parenting

As hypothesized, the results revealed that classes differed in terms of parental involvement and autonomy support. Specifically, adolescents in the *Communicators* class reported the highest levels of maternal and paternal involvement and autonomy support, whereas adolescents in the *Deceptive* class generally reported the lowest levels of these parenting dimensions. In addition, although adolescents in the *Reserved* class reported lower levels of parental involvement and autonomy support than those in the *Communicators* class, they perceived greater maternal and parental involvement as well as greater maternal autonomy support than those in the *Deceptive* class. These findings are consistent with previous studies showing that adolescents who are more likely to disclose information to their parents about their daily life are those whose parents are responsive (e.g., Soenens et al., 2006) and autonomy-supportive (e.g., Mageau et al., 2017). Hence, adolescents play an active role in the socialization process through their evaluations and responses to parental behaviors (Soenens & Vansteenkiste, 2020).

One possible way to explain adolescents’ information management strategies used to respond to their parents’ behaviors is through the lens of their needs for autonomy and relatedness as defined in SDT (Ryan & Deci, 2017). The perception of parents’ behaviors as satisfying (vs. thwarting) their needs is indeed likely to influence adolescents’ likelihood to disclose (vs. conceal) information to maintain (or reaffirm) a sufficient level of need satisfaction in the relationship with their parents (Wuyts et al., 2018). In cases where the relationship with their parents are perceived by adolescents as uninvolved and unsupportive of autonomy (similar to those in the *Deceptive* class) and, therefore, threatening their autonomy, the use of concealment strategies could be interpreted as a way of expressing autonomy in the relationship with their parents (Parkin & Kuczynski, 2012). Similarly, it is also possible that these adolescents perceived their parents’ behaviors as compromising their relatedness need and, in turn, actively hide information from their parents because they want to protect family relationships. These adolescents could also feel disengaged or detached from their parents and strongly value independence (Van Petegem et al., 2013). This echoes the fact that adolescents who are concerned about maintaining privacy over personal information (Tilton-Weaver & Marshall, 2008), negative parental reactions (e.g., sanctions, worries, or disappointments; Metzger et al., 2020), or lack of parental interest (Fletcher & Blair, 2018) are more likely to withhold information. Conversely, for adolescents who perceive their relationship with their parents as need-supportive (such as those in the *Communicators* class), they attempt to maintain an intimate relationship with their parents through disclosure and share information for certain purposes, and thus preserve both their needs for relatedness and autonomy (Tokić Milaković et al., 2018). This echoes adolescents’ reasoning for informing their parents that includes building or maintaining trust (Bakken & Brown, 2010) or positive parental reactions (e.g., empathic understanding, support; Tokić & Pećnik, 2011).

As for the dimension of parental structure, the results are more mixed. Indeed, the classes differed only in terms of paternal structure. It may be important to consider the communication style and the general family climate in which parental rule setting (Van Petegem et al., 2017) and monitoring practices (Baudat et al., 2020) take place. Indeed, when parents are only moderately involved and autonomy-supportive, their limits and regulations may be perceived and appraised differently, compared to when parents are high on involvement and autonomy support, hence bringing about adolescents to employ different communication management strategies. In line with this, the present findings suggest that between-class differences are particularly pronounced for autonomy support and involvement, and not so much for parental structure in the context of adolescents’ disclosure.

### Information Management Classes and Autonomous Reasons for Disclosure

As expected, the results indicated that information management classes differed in terms of adolescents’ autonomous reasons disclosing information to parents. Specifically, adolescents in the *Communicators* class reported the highest levels of autonomous reasons, whereas adolescents in the *Deceptive* class reported the lowest levels. These findings are consistent with the observed between-class differences in parental involvement and autonomy support. In addition to reporting the highest levels of parental involvement and autonomy support, adolescents in the *Communicators* class are likely to share information because they personally choose to do so. In contrast, adolescents in the *Deceptive* class who report lower levels of parental involvement and autonomy support would share information because they feel forced. In this case, withholding information for autonomous reasons might actually be considered a more adaptative response strategy to parental behaviors that may frustrate their needs (e.g., controlling or intrusive practices), as compared to the disclosure of information for controlled reasons (Vansteenkiste, 2018). Indeed, disclosing information under parental pressure can be seen as a form of rigid compliance, a response that occurs when adolescents submit to need-thwarting parenting behaviors because they feel pressured to do so (Soenens et al., 2015). This type of response is considered less optimal, as adolescents completely set aside their preferences and choices, and which has been shown to predict internalizing difficulties (Brenning et al., 2019).

Overall, these findings are consistent with previous studies indicating that adolescents interpret parental behaviors in terms of meeting their basic psychological needs, which, when supported, increases their disclosure (Tokić Milaković et al., 2018) and autonomous reasons for that disclosure (Wuyts et al., 2018). Therefore, it seems important that both parents provide a responsive and autonomy-supportive context and refrain from using controlling practices, as the latter is likely to be ineffective or even backfire, bringing more secrets and lies instead of disclosure (Baudat et al., 2020).

### Information Management Classes and Problematic Alcohol Use

As hypothesized, the results indicate that classes of information management were associated with problematic drinking among adolescents. Specifically, adolescents in the *Communicators* class reported the least problematic drinking, whereas adolescents in the *Deceptive* class reported the most problematic drinking. In addition, although adolescents in the *Reserved* class reported more problematic alcohol use than adolescents in the *Communicators* class, they were less involved in such behaviors than adolescents in the *Deceptive* class, one in two of whom were concerned with problematic drinking patterns. Consistent with previous studies examining the relationship between patterns of information management and adolescent adjustment (Cumsille et al., 2010) and well-being (Elsharnouby & Dost-Gözkan, 2020), it appears that it is particularly those adolescents who frequently withhold information from their parents (by keeping secrets or lying) and at the same time sporadically only disclose (i.e., those in the *Deceptive* class) who have the most drinking problems. In contrast, adolescents who frequently disclose are less affected by these problems—and this is even though they sometimes keep secrets. The latter point provides a more nuanced picture of concealment, which has mostly been viewed as the dark side of communication because of its negative associations with adolescent development. These strategies do not consistently have a negative impact on adolescent adjustment if they are only used occasionally by adolescents and accompanied by disclosure to both parents (Talwar & Crossman, 2011).

Because of the cross-sectional nature of the links between class membership and problem drinking, they could be interpreted in two ways. On the one hand, information management patterns could be symptomatic of problematic drinking behaviors. When adolescents decide to disclose or withhold information, they typically consider their own behaviors (Marshall et al., 2005). For example, adolescents who engage in drinking behaviors generally prefer to disclose less information and withhold information to avoid negative parental reactions, such as punishment or parental disappointment (Metzger et al., 2020). On the other hand, specific information management strategies may also be predictive of changing drinking patterns. Indeed, past research found that disclosure is associated with adolescents’ substance use through parental knowledge (e.g., Kapetanovic et al., 2020). Given that adolescents’ disclosures are the primary source of parents’ knowledge (Kerr et al., 2010), parents whose adolescents disclose less and retain more information have less knowledge about their children’s daily activities, whereabouts, and friends. In turn, parents with limited information brought to their attention have fewer opportunities to guide their children (Marshall et al., 2005) or regulate their potential relationships with deviant peers (Laird et al., 2008). Ultimately, the nature of the relationship between information management and alcohol use is likely to be bidirectional and may potentially bring about a vicious cycle: adolescents who drink alcohol more frequently are more likely to hide information, which may yield further increases in their alcohol consumption (McCann et al., 2016). From a prevention point of view, it is therefore important for parents to offer a supportive family climate that encourages adolescents to continue sharing information with their parents, even when they engage in risky behaviors such as alcohol use.

### Limitations and Future Directions

Despite its strengths, including the use of a person-centered approach to identify information management profiles of adolescents in the relationship with their mother and their father, this study has several limitations that could be addressed in future studies. An initial limitation concerns the measures used. Although we sought to examine associations between adolescents’ information management classes and alcohol use, the measures of information management strategies covered a limited number of topics, such as school or leisure activities. However, based on social domain theory (Nucci, 1996; Smetana et al., 2014; Turiel, 1983), previous research has shown that adolescents manage parental knowledge about their daily lives differently depending on whether they feel parental authority is legitimate over the topic of discussion (e.g., Smetana & Metzger, 2008). Future research would benefit from developing questionnaires specific to the psychosocial adjustment indicator under consideration, and to consider whether or not information is considered private or not based on adolescents’ perception. Similarly, of the parenting measures we used, only one was specific to the context of adolescent information management. Although the results showed that adolescents disclose information more frequently in responsive and autonomy supportive parenting contexts, existing measures assessing parenting that directly address adolescents’ information management (e.g., Tokić Milaković & Pećnik, 2014) should be more widely used. In addition, to get a complete picture of parental dimensions related to adolescent information management, future studies could also consider need-thwarting parenting (Vansteenkiste & Ryan, 2013), such as parental overprotection (e.g., Van Petegem et al., 2020). Second, for each type of strategy, we combined several items to obtain an overall frequency of use of that strategy. Indeed, the topic of discussion covered by the items was not systematically comparable across the strategies evaluated and considering the items of each strategy separately would have introduced too much complexity in the model. In line with social domain theory (Nucci, 1996; Smetana et al., 2014; Turiel, 1983), it is also an important avenue of research to examine the frequency of use of information management strategies that cover similar topics of discussion in order to explore and compare what information is disclosed, omitted (by secrets), or distorted (by lies). Third, because the adolescents in the sample had two parental figures, regardless of the biological nature of that relationship, the results may not be representative of families with only one parental figure or rainbow families. Finally, the cross-sectional design of the study does not allow us to infer the direction of effects between adolescents’ information management strategies, parenting and alcohol use, nor does it allow us to examine whether class membership changes over the course of adolescence. Future longitudinal studies are needed to examine the development of information management patterns during adolescence, because what adolescents consider private information and how they manage this information is supposed to change with age (Smetana et al., 2006).

## Conclusion

Adolescents’ use of disclosure and concealment strategies in the relationship with their parents have important implications for their adjustment. However, as far as we know, the patterns of disclosure, secrecy, and lying to mothers and fathers, and their relations with parenting, motivation to disclose, and alcohol use have not yet been examined. Using latent class analysis, this study filled this gap by describing constellations of information management among adolescents based on frequency of use of disclosure, secrets, and lies with their mothers and their fathers, and exploring how these patterns differ in terms of perceived need-supportive parenting, autonomous reasons for disclosure, and problematic alcohol use. As expected, the findings highlighted the heterogeneity of the information management process among middle adolescents. Specifically, three unique patterns of information management in adolescents emerged: *Reserved*, *Communicators*, and *Deceptive*. Communication remained relatively open in most families in which parents are perceived as need-supportive and adolescents report less problematic drinking. Communication was more limited among adolescents who reported perceiving the lower levels of parental involvement and autonomy support as well as less autonomous reasons for disclosure, and those who engaged more often in problem drinking. These findings highlight the importance for adolescents to continue sharing information with both parents, even if they also sometimes keep secrets, and for parents to offer an atmosphere that encourages adolescents to freely talk to them.
